# Longitudinal Analysis of HIV-2 Proviral DNA Reveals Archived Protease Inhibitor Resistance and Reservoir Evolution over Eight Years

**DOI:** 10.3390/ijms27125183

**Published:** 2026-06-08

**Authors:** Paloma Gonçalves, Inês Lopes, Andreia Martins, Filipa Maia, Francisco Martin, Pedro Borrego, Francisco Antunes, Emília Valadas, Claudia Palladino, Inês Bártolo, Nuno Taveira

**Affiliations:** 1 Research Institute for Medicines (iMed.ULisboa), Faculty of Pharmacy, Universidade de Lisboa, 1649-003 Lisboa, Portugal; pcb.goncalves@campus.fct.unl.pt (P.G.); is.lopes@campus.fct.unl.pt (I.L.); andreiardgmartins@gmail.com (A.M.); filipa14maia@gmail.com (F.M.); rickmartin_5@hotmail.com (F.M.); palladino.claudia@gmail.com (C.P.); ibartolo@ff.ulisboa.pt (I.B.); 2 Centro de Administração e Políticas Públicas (CAPP), Instituto Superior de Ciências Sociais e Políticas, Universidade de Lisboa, 1300-663 Lisbon, Portugal; pborrego65@gmail.com; 3 Instituto de Saúde Ambiental, Faculdade de Medicina da Universidade de Lisboa and Laboratório Associado TERRA, Universidade de Lisboa, 1300-663 Lisbon, Portugal; fantunes@medicina.ulisboa.pt; 4 Serviço de Doenças Infeciosas, Faculdade de Medicina de Lisboa, Hospital de Santa Maria, 1649-035 Lisboa, Portugal; evaladas@medicina.ulisboa.pt; 5 CE3C-Centre for Ecology, Evolution and Environmental Changes, Faculdade de Ciências, Universidade de Lisboa, 1749-016 Lisboa, Portugal; 6 Centro de Investigação Interdisciplinar Egas Moniz (CiiEM), Egas Moniz School of Health and Science, Monte de Caparica, 2829-511 Caparica, Portugal

**Keywords:** HIV-2, protease inhibitor resistance, proviral DNA sequencing, antiretroviral therapy, viral reservoirs

## Abstract

Protease inhibitors (PIs) remain important components of HIV-2 treatment, but resistance genotyping is frequently challenging in individuals with low or undetectable plasma viremia. Proviral DNA sequencing may provide access to archived viral variants and improve understanding of long-term resistance and clinical evolution. In this retrospective longitudinal study, 27 individuals with HIV-2, both ART-experienced and ART-naïve, followed at a hospital in Lisbon, were analyzed. The HIV-2 protease gene was amplified from peripheral blood mononuclear cell-derived proviral DNA, cloned, and sequenced (Sanger sequencing) at baseline and, for ART-treated participants, after eight years of follow-up. Resistance profiles were interpreted using the Stanford HIVdb, HIV-2EU, and Rega algorithms. Clinical data, including ART history, CD4 counts, and plasma viral load, were collected longitudinally. Amino acid diversity was assessed using Shannon entropy, and longitudinal CD4 dynamics were evaluated using mixed-effects models with time-varying ART exposure. Sensitivity analyses were performed using generalized estimating equations (GEE). A total of 222 clonal HIV-2 protease sequences clustered within group A. Major PI resistance mutations were detected in 21.4% of ART-experienced and 23.1% of ART-naïve individuals at baseline. Longitudinal resistance trajectories varied across participants, including persistence, apparent emergence, and non-detection of previously identified mutations. Mixed-effects modeling revealed substantial inter-individual variability in CD4 trajectories, with no statistically significant associations observed between CD4 evolution and ART status, time, or their interaction. GEE analyses yielded consistent results, supporting robustness across modeling frameworks. Entropy analysis identified localized sequence diversity changes restricted to a small number of protease residues, with positions 60 and 75 differing between groups at baseline and position 21 showing longitudinal variation among treated participants. This study demonstrates that proviral DNA sequencing captures archived HIV-2 protease diversity and reveals persistent and dynamic resistance patterns within the viral reservoir. While no population-level association between ART exposure and CD4 trajectory was observed, marked inter-individual variability highlights the complexity of longitudinal immune recovery in HIV-2 infection. These findings support the value of proviral sequencing as a complementary research tool for characterizing long-term viral evolution in settings where plasma-based genotyping is limited.

## 1. Introduction

Human immunodeficiency virus type 2 (HIV-2) is predominantly endemic in West Africa, with significant prevalence rates in countries such as Guinea-Bissau, Senegal, and Cape Verde [1,2,3]. Due to historical and migratory ties, Portugal has the highest number of people living with HIV-2 outside of West Africa [4]. In 2023, HIV-2 was responsible for 3% of all HIV infections in the country [5]. A total of 2171 HIV-2 infection cases were reported in Portugal from 1983 to 2023. Of these, 698 progressed to an AIDS diagnosis, with 588 resulting in death. The median age at death was 62 years, and the mean interval between HIV-2 diagnosis and death was approximately 10 years [5].

HIV-2 is classified into nine phylogenetic groups (A–I), although only groups A and B have demonstrated epidemic spread in humans [6]. Group A predominates in West Africa and has been reported as the principal circulating group in Portugal [4,7].

HIV-2 is associated with slower disease progression, lower transmissibility, and lower plasma viral loads (VL) compared to HIV-1 [8,9]. Nonetheless, without effective antiretroviral therapy (ART), HIV-2 infection can ultimately lead to acquired immunodeficiency syndrome (AIDS)-related morbidity and mortality [9,10,11]. The management of HIV-2 infection poses distinctive challenges due to the intrinsic resistance to non-nucleoside reverse transcriptase inhibitors, reduced susceptibility to certain protease inhibitors (PIs), and limited therapeutic options overall [12,13,14,15]. Current HIV-2 treatment relies primarily on integrase strand transfer inhibitors, with selected protease inhibitors and nucleoside reverse transcriptase inhibitors remaining essential components of effective regimens [16]. The limited availability of clinical trials, standardized resistance interpretation algorithms, and validated diagnostic tools for HIV-2 restricts the implementation of individualized treatment strategies, particularly in resource-limited settings where HIV-2 is most prevalent [12,17,18,19,20]. Ensuring long-term treatment efficacy requires continuous monitoring, access to second-line therapies, and global efforts to improve drug availability and resistance surveillance.

The World Health Organization (WHO) has issued guidelines recommending the implementation of HIV-2 RNA-based genotyping as a method for detecting drug resistance mutations [21]. However, RNA-based testing can pose significant challenges in the context of HIV-2 infection, particularly in cases where VL is low or undetectable. In such cases, proviral DNA genotyping provides a valuable alternative, allowing the identification of archived resistance mutations [22,23,24]. In addition, clonal sequencing improves the resolution of resistance testing by enabling the detection of minority variants that may influence treatment outcomes and contribute to the evolution of drug resistance over time [25,26,27].

This eight-year retrospective longitudinal study of a Portuguese HIV-2 cohort explored the potential of proviral DNA as a tool for investigating archived resistance mutations and long-term clinical and virological evolution. By analyzing peripheral blood mononuclear cell (PBMC)-derived proviral reservoirs, we evaluated resistance patterns, sequence diversity, and longitudinal clinical trajectories in individuals with predominantly low or undetectable viral loads, providing insight into the long-term dynamics of HIV-2 infection during antiretroviral therapy.

## 2. Results

### 2.1. Study Population

Twenty-seven PLWHIV-2 were enrolled in this study (Table 1). The median age of the participants was 49 years (interquartile range, IQR: 27–64 years), and the majority (70%) were female. Of these, 14 (52%) were ART-experienced and 13 (48%) were ART-naïve. Sex, age, and country of origin were not associated with ART exposure status (*p* > 0.05). Baseline CD4 counts differed significantly between ART groups in univariate analysis (Mann–Whitney U test: W = 57, *p* = 0.0015), with lower values observed in the treated group. Of the ART-experienced participants (n = 13, 93%), all but one were on a baseline regimen comprising a boosted PI (2NRTI + LPV/r, IDV/r or SQV/r; NRTI + RAL + DRV/r). Six participants on ART were severely immunosuppressed at baseline, with a CD4+ T cell count of less than 200 cells/μL (range 72–190 cells/μL). Five patients had no baseline VL data. Of the remaining participants, 17 (63%) had an undetectable VL: eight (62%) of those who were ART-naïve and nine (64%) of those on ART. Overall, three patients died and one was lost to follow-up in the ART-experienced group, and six patients were lost to follow-up in the ART-naïve group. An additional death occurred among those lost to follow-up.

### 2.2. Follow-Up of Clinical Outcomes

The longitudinal trend of CD4^+^ T cell counts and VL among participants in this study is shown in Tables S1–S3.

**Figure 1:**
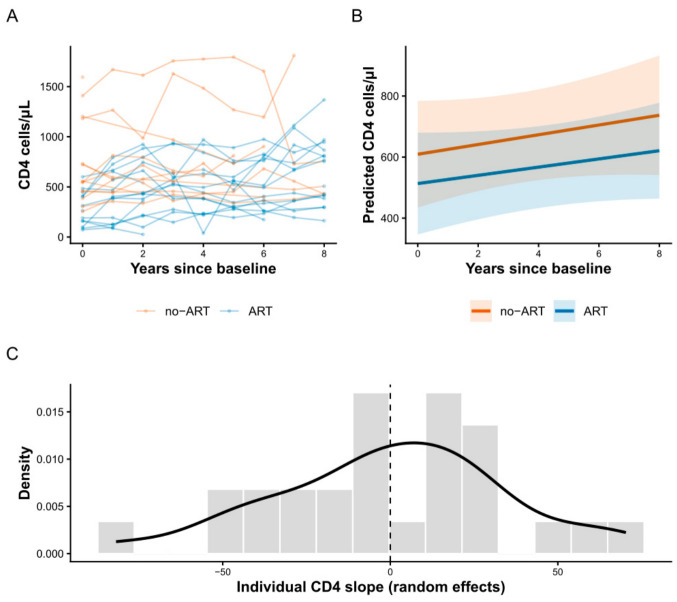
Longitudinal CD4 trajectories and inter-individual variability assessed using linear mixed-effects models. (**A**) Individual CD4 trajectories over time for each participant, stratified by antiretroviral treatment (ART) status. Each line represents repeated measurements from a single individual. (**B**) CD4 trajectories estimated using a linear mixed-effects model including ART status, time since baseline, and their interaction, with patient-level random intercepts and slopes. Solid lines represent model-based predicted trajectories and shaded areas indicate 95% confidence intervals; points represent observed group means. No statistically significant effects of time, ART status, or their interaction were observed in the model. (**C**) Distribution of patient-specific random slope estimates from the mixed-effects model, illustrating inter-individual variability in CD4 trajectories over time.

Longitudinal CD4 dynamics were evaluated using linear mixed-effects models incorporating repeated observations within individuals and treating ART exposure as a time-varying covariate throughout follow-up (Figure 1). Two model structures were compared: a random-intercept model and a random-intercept plus random-slope model. Model comparison demonstrated that inclusion of patient-specific slopes significantly improved model fit (AIC = 2464 vs. 2490; likelihood ratio test χ^2^ = 28.1, df = 2, *p* < 0.001), indicating substantial inter-individual variability in longitudinal CD4 trajectories.

In the selected random-slope model, the estimated baseline mean CD4 count for the reference (ART-naïve) group was approximately 609 cells/µL (*p* < 0.001). In contrast to the univariate analysis (Table 1), no statistically significant associations were observed for ART status (β = −96.0, *p* = 0.233), follow-up time (β = 16.0 cells/year, *p* = 0.265), or the ART-by-time interaction (β = −2.5 cells/year, *p* = 0.870), indicating no evidence of differential CD4 trajectories according to treatment status at the population level.

Random-effects estimates demonstrated marked between-patient heterogeneity in both baseline CD4 levels and temporal slopes (Figure 1). As a sensitivity analysis, generalized estimating equations (GEE) with an exchangeable correlation structure were also fitted to assess population-averaged longitudinal effects. Consistent with the mixed-effects model results, no statistically significant associations were observed for ART status (*p* = 0.111), follow-up time (*p* = 0.743), or the ART-by-time interaction (*p* = 0.161), supporting the robustness of the findings across alternative longitudinal modeling approaches.

Regarding VL, the proportion of participants with undetectable HIV-2 RNA remained high throughout the follow-up period, ranging from 38% to 64% across different years. However, a subset of participants continued to exhibit detectable VL (>40 RNA copies/mL), with proportions varying between 14% and 33% over time, highlighting potential virologic failure. It should be noted that VL data availability decreased at later timepoints, particularly in the final year of follow-up.

### 2.3. HIV-2 Genetic Diversity

A total of 89 clonal sequences were obtained from ART-experienced participants and 100 from ART-naïve participants at baseline (median: 8 clones/participant; min–max: 1–11) (Table S4). Thirty-three clonal sequences were obtained from the samples collected on the final year of the study, all from ART-experienced participants (median: 3 clones/participant; min–max: 0–9) (Table S5). Screening for APOBEC-associated G-to-A hypermutation revealed no hypermutated HIV-2 protease sequences, and all sequences were retained for subsequent analyses.

**Figure 2:**
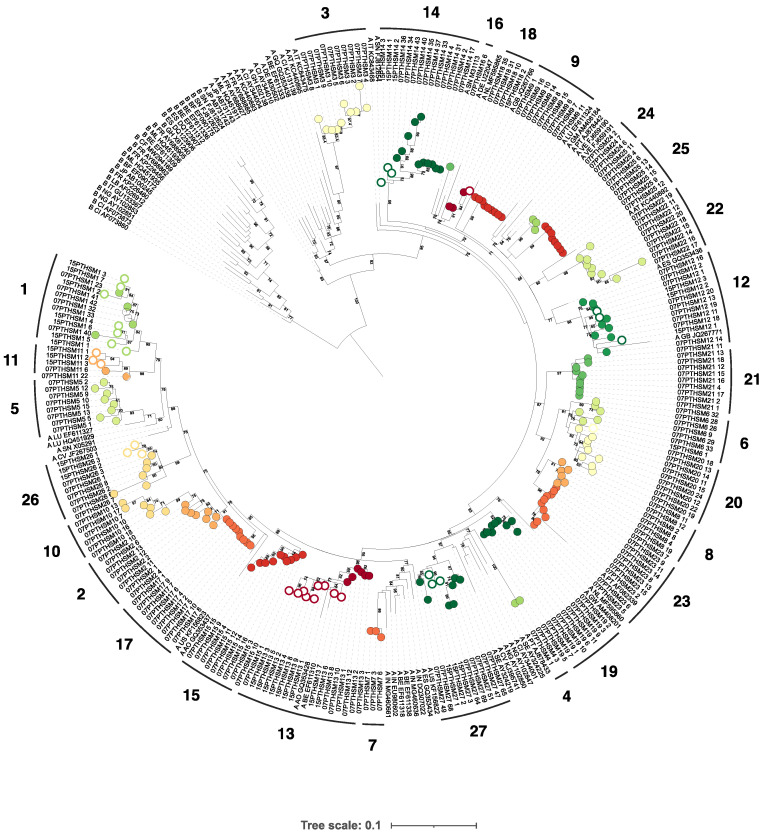
Maximum likelihood phylogenetic tree analysis of HIV-2 protease sequences. Alignment of PR nucleotide sequences was used to construct a maximum likelihood tree under the TIM2+F+R4 substitution model. HIV-2 reference sequences from different locations were also used. Clonal PR sequences (PTHSM1–PTHSM27) are associated with the participant number next to the respective cluster. The clonal PR sequences from baseline are represented by filled circles, while those from year 8 are represented by open circles. Sequences without symbols correspond to HIV-2 reference sequences. Tree scale is number of substitutions per site. The country codes are based on ISO 3166-1 alpha-2 code. Bootstrap values at least 70% are shown.

All sequences analyzed were HIV-2 group A (Figure 2). Clonal sequences consistently formed participant-specific monophyletic clades, with no evidence of cross-participant clustering. The mean intra-patient genetic distance was 0.021 ± 0.012 substitutions per site, with no significant difference between ART-experienced and ART-naïve participants (0.02447 vs. 0.01587, *p* = 0.100). No evidence of epidemiological linkage between participants was found, except for PTHSM8 and PTHSM23, who were identified as a couple.

### 2.4. Amino Acid Diversity Analysis

**Figure 3:**
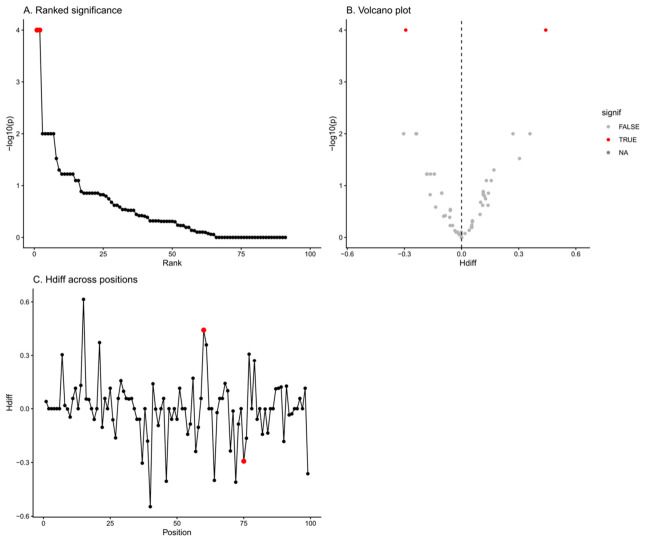
Site-specific entropy differences in HIV-2 protease sequence of naïve (background dataset) and ART treated individuals (query dataset) at baseline. (**A**) Ranked significance of amino acid positions based on −log10(*p*-values) derived from permutation testing (10,000 randomizations). Positions are ordered from most to least significant, with residues 60 and 75 highlighted in red. (**B**) Volcano plot showing entropy differences between PR sequences from treated and naïve individuals (Hdiff) versus statistical significance (−log10 *p*-value). The horizontal dashed line indicates the nominal significance threshold (*p* = 0.05), and vertical line marks no difference in entropy (Hdiff = 0). Significant positions after false discovery rate (FDR) correction are shown in red. (**C**) Distribution of entropy differences (Hdiff) across the protein sequence by residue position. Positive values indicate higher variability in the query set (treated) relative to background (naïve), while negative values indicate reduced variability. Residues 60 and 75 show the strongest and most statistically robust deviations and remain significant after Benjamini–Hochberg FDR correction (FDR < 0.05).

Site-specific entropy analysis of HIV-2 protease sequences identified localized differences in amino acid diversity between treated and treatment-naïve individuals at baseline. Across the protease sequence, most residues showed no significant entropy differences after correction for multiple testing, indicating that sequence variability was largely conserved and restricted to a small number of discrete sites rather than broadly distributed along the protein (Figure 3).

Among the 99 amino acid positions analyzed, residues 60 and 75 displayed the strongest deviations from the null distribution, each reaching the minimum empirical *p*-value attainable with the permutation framework (*p* ≈ 1 × 10^−4^) (Table S4). Both positions remained significant following Benjamini–Hochberg correction (FDR = 0.005). Residue 60 (lysine in the treated consensus sequence) exhibited increased diversity in treated individuals relative to treatment-naïve patients (Hdiff = 0.442), whereas residue 75 (isoleucine) showed reduced diversity (Hdiff = −0.293), indicating opposing patterns of sequence variability at these positions.

To investigate the longitudinal effect of treatment, protease diversity was further compared between baseline and study-end sequences obtained exclusively from treated individuals (Table S5). In contrast to the baseline comparison, a single residue remained significant after FDR correction: position 21 (FDR = 0.010; Figure S1). Position 21 (glutamic acid in the study-end consensus sequence) exhibited reduced variability at study end relative to baseline (Hdiff = −0.363), suggesting increased sequence conservation over prolonged treatment exposure.

Together, these findings indicate that protease evolution under antiretroviral exposure is characterized by site-specific shifts in sequence diversity rather than widespread changes across the protein. The distinct signatures observed in the cross-sectional and longitudinal analyses further suggest that baseline treatment status and long-term treatment exposure may exert different selective pressures on HIV-2 protease evolution.

### 2.5. Drug Resistance Mutations

**Figure 4:**
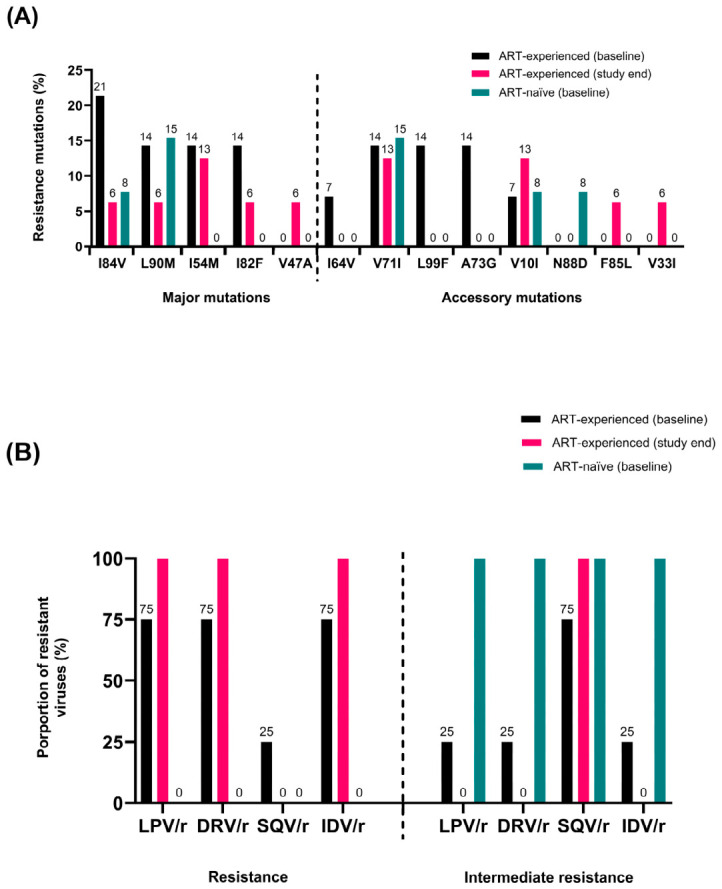
Genotypic and estimated phenotypic resistance. (**A**) Frequency of PI resistance-associated mutations in the study population. Results are shown separately for ART-experienced and ART-naïve participants and further divided by study timepoint (study entry vs. study end in year 8). (**B**) Level of resistance to each drug among participants with resistance mutations as assessed using HIV-2EU 4. Results are shown separately for ART-experienced and ART-naïve participants and further divided by study timepoint (baseline vs. study end in year 8). The figure is intended as a descriptive and exploratory visualization of temporal patterns rather than a formal paired comparison between baseline and year-8 resistance profiles. Longitudinal interpretation should be performed cautiously because follow-up data were incomplete for some participants and the number of clones analyzed differed across time points. Drug abbreviations: IDV/r—indinavir/ritonavir; SQV/r—saquinavir/ritonavir; DRV/r—darunavir/ritonavir; LPV/r—lopinavir/ritonavir.

At baseline, protease mutations associated with PI resistance (major and/or accessory) were slightly more frequent in ART-experienced participants (6/14, 43%) than in ART-naïve participants (5/13, 38%) (Figure 4A; Tables S6 and S8). Four ART-experienced individuals (21.4%) carried major resistance mutations (PTHSM1, PTHSM3, PTHSM4, PTHSM11) with mutations fixed in the viral population in two participants and mutations present at lower frequencies in the remaining participants (≤50% of clones). The most frequent major resistance mutations among these participants were I54M, I82F, I84V, and L90M, present in two (14.3%) participants each. Accessory resistance mutations were more common, detected in all but one ART-experienced participant (83.3%), with three individuals showing fixation across all clones.

Among the ART-naïve participants, three individuals (23.1%) carried major resistance mutations. Here, however, major mutations never exceeded 36.4% of clones, while accessory mutations displayed striking heterogeneity, ranging from rare (1% of clones) to fixed (100%). Among these participants, L90M was the most frequent major resistance mutation (15%), followed by I84V (8%).

By year 8, all participants with available clonal sequences (n = 9) were receiving antiretroviral therapy (ART), either from the start of the study (n = 6) or after initiating treatment between two- and eight-years post-enrollment (n = 3). Two individuals, PTHSM11 and PTHSM12, carried two to three major resistance mutations, with I54M being the most common, while PTHSM18 acquired an accessory resistance mutation (V10I) (Figure 4A; Tables S7 and S8). While PTHSM11 showed a doubling of major resistance mutations compared to study entry, no resistance mutations were present at baseline in PTHSM12 and PTHSM18. Major (PTHSM1, PTHSM14) or accessory (PTHSM27) resistance mutations detected at baseline were not detected at study end in three participants (33.3%). Finally, participants PTHSM6, PTHSM13, and PTHSM26 remained free of resistance mutations throughout the study.

Overall, the longitudinal patterns observed across participants highlight substantial heterogeneity, including persistence, apparent emergence, and non-detection of previously identified mutations during ART. Despite this variability, a subset of participants-maintained mutation-free profiles, underscoring the potential for durable viral suppression under effective therapy.

### 2.6. Impact of PI Resistance-Associated Mutations

Of the four ART-experienced individuals who showed resistance mutations at the start of the study, three (PTHSM3, PTHSM4 and PTHSM11) were resistant or intermediate resistant to all five PIs (Figure 4B). By the end of the study, both participants with resistance mutations (PTHSM11 and PTHSM12) exhibited resistance or intermediate resistance to all PIs. The three ART-naïve individuals with drug resistance mutations (PTHSM14, PTHSM23 and PTHSM25) showed intermediate resistance to all PIs.

The clinical impact of PI resistance was evident in both PTHSM3 and PTHSM4, who died during the study. At the start of the study, both individuals exhibited viral loads (VLs) of >100,000 and 75,571 HIV-2 RNA copies/mL, respectively. PTHSM3, who was receiving DRV/r-based treatment, harbored mutations I84V and L90M, which are associated with resistance to this drug [28], in all proviral DNA clonal sequences obtained at baseline (Tables S6 and S8). The VL remained detectable one year later, accompanied by persistently low CD4^+^ T cell counts, and the individual subsequently died. PTHSM4, who was initially on an LPV/r-based regimen, harbored I54M, I82F and L90M mutations in all baseline proviral DNA clones, which confer resistance to all PIs [28]. Despite transitioning to an SQV/r-based regimen, the individual experienced continued immunological decline and died two years after study entry, consistent with treatment failure.

Participants PTHSM1 and PTHSM14 lost their major resistance mutations (I84V and L90M, respectively) during the follow-up period (Table S7). PTHSM14 started treatment in the fifth year of the study, taking DRV/r + RAL, which led to undetectable VL and complete immunological recovery with a CD4+ T cell count of 1369 cells/mm^3^ at the end of the study. Similarly, PTHSM1 maintained an undetectable VL and elevated CD4+ T cell counts throughout the study period with an LPV/r-based regimen.

PTHSM12 exhibited fluctuating CD4^+^ T cell counts and VL throughout the study. The participant was initially on an SQV/r-based regimen, which was switched to LPV/r in year 2 and subsequently to a DRV/r-based regimen from year 3 to 8. In the final genotypic analysis, resistance mutations I54M and I84V were identified in one clone, both associated with resistance to DRV/r [28]. Despite low CD4^+^ T cell counts (163 cells/mm^3^), the participant completed the study with a suppressed VL.

PTHSM11 was taking an LPV/r-based regimen at baseline and exhibited major resistance mutations (I54M, I82F) in one of two sequenced clones (Tables S6 and S8). In response to a high VL (9309 HIV-2 copies/mL) and decreasing number of CD4+ T cells (from 161 to 124 cells/μL) at year 1, treatment was switched to an SQV/r-based regimen, followed by a transition to DRV/r-based regimens in year 3. By year 8, the patient had achieved virologic suppression and CD4+T cells rose to 420 cells/μL; however, two additional resistance mutations, L90M and V47A, were detected, neither of which had been present at baseline (Table S7). The final mutation profile of this participant is usually associated with resistance to all PIs [28].

## 3. Discussion

This study provides longitudinal insight into HIV-2 genetic diversity, archived drug resistance, and clinical evolution in a Portuguese cohort followed over eight years. Using proviral DNA clonal sequencing, we identified resistance-associated mutations in both ART-naïve and ART-experienced individuals and evaluated their relationship with long-term clinical and evolutionary patterns. These findings support the utility of proviral DNA genotyping for studying resistance in individuals with low or undetectable plasma viral loads.

Longitudinal CD4 analysis incorporating time-updated ART exposure demonstrated substantial between-patient variability in both baseline values and temporal trajectories. Model comparison strongly supported inclusion of patient-specific slopes, indicating marked inter-individual heterogeneity in CD4 evolution over time. Although no statistically significant associations were observed for ART status, follow-up time, or their interaction in the mixed-effects model, these findings were consistent with results from sensitivity analyses using generalized estimating equations (GEE), which also showed no significant population-averaged effects of ART exposure or time. Similar heterogeneity in longitudinal CD4 trajectories has been reported in HIV-1 cohorts, where substantial inter-individual variation persisted despite long-term antiretroviral therapy and virological suppression [29]. In addition, HIV-2 infection has been associated with heterogeneous immunological responses and slower or less predictable CD4 recovery patterns compared with HIV-1 [30]. Notably, participants receiving ART in our cohort had substantially lower baseline CD4 counts than untreated individuals, suggesting differences in disease stage at treatment initiation that may have contributed to divergent immunological trajectories. Overall, these findings suggest that CD4 dynamics in this cohort may be primarily driven by individual-level factors rather than by uniform, treatment-associated trends. This heterogeneity could be due to differences in treatment history, adherence, infection duration, host characteristics or baseline immune status that are not accounted for in the current model.

Site-specific entropy analysis showed that sequence diversity differences between ART-experienced and ART-naïve individuals were restricted to a small number of discrete protease residues rather than distributed broadly across the protein. After correction for multiple testing, only positions 60 and 75 remained significant. Position 60 exhibited increased diversity in treated individuals, whereas position 75 showed reduced diversity, indicating distinct directions of entropy shift. In the longitudinal comparison between baseline and study-end samples from treated participants, only residue 21 remained significant after FDR correction. Although these positions are not currently recognized as major HIV-2 PI resistance sites [12,31], their localized changes in diversity may reflect treatment-associated evolutionary processes or selective pressures acting on the viral reservoir.

Importantly, 21.4% of ART-experienced individuals and 23.1% of ART-naïve individuals carried at least one proviral clone containing major PI resistance mutations at baseline. The most frequently observed mutations included I54M, I82F, I84V, and L90M, which have been associated with resistance to clinically relevant HIV-2 protease inhibitors [12,31]. While directly comparable proviral HIV-2 datasets are limited, previous studies have demonstrated persistence of archived resistance mutations in proviral reservoirs despite sustained virological suppression [12]. The unexpectedly high prevalence of resistance mutations among ART-naïve participants may reflect transmitted resistance or undocumented prior treatment exposure and highlights the potential clinical relevance of baseline resistance assessment [21,32,33].

Longitudinally, resistance trajectories varied across participants, including persistence, apparent emergence, and non-detection of previously identified mutations. Detection of resistance-associated mutations in ART-treated individuals with sustained virologic suppression (e.g., PTHSM11 and PTHSM12) suggests that these variants may persist within the proviral reservoir over extended periods despite effective treatment [34,35,36,37,38,39]. However, absence of a previously detected mutation cannot be interpreted as definitive loss of that variant because follow-up sequence numbers were lower and clone sampling depth varied across timepoints. Therefore, these findings should be interpreted cautiously.

The favorable clinical outcomes observed in some participants despite detectable resistance-associated mutations may partly reflect regimen optimization during follow-up, including initiation or switching to darunavir/ritonavir-based therapy. Darunavir has a high genetic barrier to resistance, and isolated mutations such as I54M or I84V alone may not substantially reduce susceptibility [31]. Improved tolerability and adherence associated with newer regimens may also have contributed to sustained immunologic benefit. Although this cohort reflects historical treatment strategies (2007–2015), including indinavir/ritonavir and saquinavir/ritonavir, these regimens are now largely obsolete. However, several protease inhibitor resistance-associated mutations identified in this study (e.g., I54M, I82F, I84V, L90M) confer cross-resistance across the PI class, including agents still used in HIV-2 treatment such as darunavir/ritonavir. Therefore, archived resistance patterns remain relevant for understanding long-term resistance persistence and for interpreting contemporary resistance profiles.

We acknowledge that alternative sequencing strategies, particularly next-generation sequencing (NGS), offer important opportunities for studying archived HIV-2 drug resistance. NGS enables high-throughput analysis of large numbers of samples, increasing statistical power for inter-participant comparisons and supporting broader epidemiological assessments of resistance patterns [40,41,42]. At the same time, accurate haplotype reconstruction using NGS can be challenging due to higher per-base error rates, shorter read lengths, and the need for complex bioinformatic processing, which, if not carefully controlled, may inflate estimates of viral diversity [42,43]. Moreover, while NGS is now widely implemented and standardized for HIV-1 resistance testing, its application to HIV-2 remains comparatively less mature, with fewer validated assays, reference datasets, and analytical frameworks available [41,44,45]. Practical considerations, including infrastructure requirements, cost, quality assurance, and the need for specialized expertise, may also limit routine implementation in some settings [40]. In this context, we selected a cloning-based Sanger sequencing approach, which provides highly accurate, full-length reads, preserves linkage between resistance mutations, and supports robust phylogenetic reconstruction of individual proviral haplotypes. This approach is particularly well suited to longitudinal analyses of the HIV-2 reservoir, as it enables precise tracking of specific resistance variants within and between participants over time.

Several limitations should be considered. Proviral DNA may contain archived or defective genomes and therefore may not fully reflect actively replicating viral populations [46,47,48,49]. In addition, clonal sequencing and variable clone numbers may introduce sampling bias and limit detection of low-frequency variants [50,51]. The retrospective design resulted in incomplete clinical information, including limited data on duration of infection, prior ART exposure, and treatment adherence before study inclusion. Longitudinal follow-up was additionally affected by patient loss to follow-up, deaths, and incomplete viral load data at later timepoints, potentially introducing survivorship and ascertainment biases [52]. Despite these limitations, the use of longitudinally collected samples combined with haplotype-resolved proviral sequencing provides valuable insight into long-term HIV-2 reservoir evolution and resistance dynamics in a real-world clinical setting.

## 4. Materials and Methods

### 4.1. Ethics Statement

The study was approved by the Ethics Committee of the Hospital de Santa Maria, Lisbon, Portugal (Process number: 465/09), and was conducted according to the Declaration of Helsinki. Written informed consent was obtained from all the participants.

### 4.2. Study Population and Data Collection

This retrospective longitudinal study examined 27 people living with HIV-2 (PLWHIV-2) who were monitored at Hospital de Santa Maria in Lisbon, Portugal, from 2007 to 2015. Demographic characteristics, clinical outcomes, immunovirological parameters, and ART usage data were obtained from the patients’ medical records. At the beginning of the study, the participants were classified as either ART-experienced (n = 14) or ART-naïve (n = 13). Three individuals in the ART-naïve group started ART during the follow-up period. Plasma viral load was determined using an in-house test with a quantification limit of 40 HIV-2 RNA copies/mL. Viremia was categorized as either undetectable (<40 HIV-2 RNA copies/mL) or detectable (≥40 HIV-2 RNA copies/mL).

### 4.3. HIV-2 DNA Extraction

Blood samples were collected in EDTA tubes. PBMCs were isolated by centrifugation through a Ficoll-Hypaque gradient (Ficoll-Paque™ PLUS, GE Healthcare, Uppsala, Sweden). Proviral DNA was extracted from the PBMCs using the Wizard^®^ Genomic DNA Purification Kit (Promega Corporation, Madison, WI, USA), according to the manufacturer’s protocol.

### 4.4. PCR Amplification, Cloning and Sequencing

The HIV-2 protease coding region (PR) was amplified from proviral DNA using BIOTAQ™ DNA polymerase (Bioline, London, UK) and the primer set CRPR1 (5′-CCTAGAAGACAGGGMTGCTGGAA-3′; positions 2314–2336 in HIV-2ALI) and CRPR2 (5′-AGCATYCTCCATTTGTTYTTGTC-3′; positions 3148–3126). A nested PCR was then performed with a different primer set: CRPR3 (5′-TGCTGCACCTCAATTCTCTCTTTGGA-3′; positions 2624–2649) and CRPR4 (5′-TTGGTCCATCTTTYCCWGGCTT-3′; positions 2985–2964).

The thermocycling conditions consisted of an initial denaturation step at 95 °C for 2 min, followed by 40 cycles of denaturation at 94 °C for 1 min, annealing at 59 °C for 1 min and extension at 72 °C for 1 min, and a final extension step at 72 °C for 15 min. DNA amplification was performed using a Biometra T-Gradient ThermoBlock Thermocycler (Biometra, Jena, Germany). The amplified products were cloned into pCR4-TOPO^®^ (Invitrogen, Waltham, MA, USA) and sequenced using an ABI PRISM^®^ BigDye^®^ Terminator v3.1 Cycle Sequencing Kit (Applied Biosystems, Foster City, CA, USA). Sanger sequencing was performed using the primers CRPR3 and CRPR4 with an annealing temperature of 61 °C. Capillary electrophoresis was conducted on an ABI 3100-Avant Genetic Analyzer (Applied Biosystems, CA, USA).

### 4.5. HIV-2 Genotype and Phylogenetic Analysis

Protease coding nucleotide sequences were aligned to HIV-2 reference sequences obtained from the Los Alamos database (https://www.hiv.lanl.gov, accessed on 1 March 2026) using the Clustal W algorithm implemented in the ClustalX 2.1 software [53] and manual adjustments were made using Genedoc [54]. All nucleotide sequences were screened for evidence of APOBEC-mediated hypermutation using the Los Alamos Hypermut v3 tool (https://www.hiv.lanl.gov/content/sequence/HYPERMUT/hypermutv3.html, accessed on 1 March 2026) [55].

The IQ-Tree 1.6.11 software [56] was used to construct a Maximum Likelihood tree (ML) under the best fit evolutionary model TIM2+F+R4 selected using Model Finder module [57] and the Akaike information criterion, with 1000 bootstrap replicates. Clusters with bootstrap values of at least 70% were considered significant. Final visualization and annotation of the ML tree was performed using iTOLV6 (https://itol.embl.de/, accessed on 1 March 2026) [58].

### 4.6. Analysis of Drug Resistance Mutations

PI resistance-associated mutations and their estimated phenotypic impact were identified using the Stanford HIVdb Program for HIV-2 (beta) (https://hivdb.stanford.edu/hivdb/hiv2/by-sequences/, accessed on 10 March 2026) and HIV-2EU 4.0 (https://www.hiv-grade.de/HIV2EU/deployed/grade.pl?program=hivalg, accessed on 10 March 2026) [28].

### 4.7. Shannon Entropy Analysis

Site-specific amino acid diversity within HIV-2 protease (PR) sequences was quantified using Shannon entropy, where higher values indicate greater sequence variability and lower values indicate increased conservation. Entropy was calculated for each amino acid position in aligned PR sequences using the ENTROPY tool from the Los Alamos National Laboratory (LANL) HIV database. Sequences were partitioned into background and query datasets according to the comparison being performed. For each residue position, entropy values were calculated from amino acid frequency distributions within each dataset, and entropy differences (Hdiff) were computed to quantify site-specific variation between groups.

Statistical significance of entropy differences was evaluated using a permutation-based non-parametric framework. For each amino acid position, 10,000 randomized datasets were generated by permuting group labels while preserving dataset structure. Site-specific empirical null distributions of Hdiff values were constructed, and empirical *p*-values were calculated as the proportion of permutations with absolute entropy differences greater than or equal to the observed value. To account for multiple testing across amino acid positions, *p*-values were adjusted using the Benjamini–Hochberg false discovery rate (FDR) procedure [59]. Positions with FDR-adjusted *p*-values < 0.05 were considered statistically significant.

### 4.8. Statistical Analysis

Baseline demographic and clinical characteristics, including ART status, were summarized using descriptive statistics. Continuous variables are presented as median and interquartile range (IQR), while categorical variables are presented as counts and percentages. Continuous variables were compared using the Mann–Whitney U test. All statistical tests were two-sided, and *p*-values < 0.05 were considered statistically significant.

### 4.9. Longitudinal CD4 Analysis

Longitudinal CD4 dynamics were evaluated using linear mixed-effects models to account for repeated measurements within individuals over time. CD4 count was modeled as the dependent variable, with antiretroviral therapy (ART) status, time since baseline (Year_centered), and their interaction included as fixed effects. ART was treated as a time-varying covariate, updated according to documented treatment status at each follow-up visit. Time was centered at baseline (Year = 0).

To account for within-subject correlation and inter-individual heterogeneity in CD4 trajectories, patient identity was included as a random effect, with both random intercepts and random slopes for time. Model selection was performed using Akaike Information Criterion (AIC), comparing random-intercept and random-slope structures.

Sensitivity analyses were conducted using generalized estimating equations (GEE) with an exchangeable correlation structure to evaluate population-averaged longitudinal effects.

Models were fitted using restricted maximum likelihood (REML), and fixed effects were assessed using Satterthwaite’s approximation for degrees of freedom.

Longitudinal analyses were performed in R version 4.6.0 (24 April 2026) [60] using the packages lme4 [61], lmerTest [62], and nlme [63,64].

## 5. Conclusions

Proviral DNA genotyping identified archived and evolving resistance-associated mutations in HIV-2 and provided longitudinal insight into reservoir dynamics in individuals with low or undetectable viremia. These findings support the value of proviral sequencing as a complementary approach for resistance assessment and long-term monitoring of HIV-2 infection.

## Figures and Tables

**Table 1:** Demographic characteristics, clinical outcomes and immunovirological parameters of the participants at baseline.

Variable	Total (%)	ART-Experienced Participants (%)	ART-Naïve Participants (%)	*p* Value *
No. of subjects (%)	27 (100)	14 (52)	13 (48)	
Sex [n (%)]				0.6957
Female	19 (70)	10 (67)	9 (75)	
Male	8 (30)	5 (33)	3 (25)	
Age (years), [median (IQR)]	49 (37–58)	48 (40–57)	49 (33–60)	1.0000
Country of origin [n (%)]				
Portugal	8 (30)	5 (33)	3 (25)	0.6957
Guinea-Bissau	12 (45)	5 (33)	7 (58)	0.2576
Cape Verde	3 (11)	3 (20)	0 (0)	0.2308
Mozambique	2 (7)	2 (14)	0 (0)	0.4872
Unknown	2 (7)	0 (0)	2 (17)	0.1880
Median CD4^+^ T cell count (cells/μL), [median (IQR)]	448 (259–579)	264 (159–409)	552 (455–1069)	0.0015
Viral load, category [n (%)]				
Undetectable (<40 RNA copies/mL)	17 (63)	9 (64)	8 (62)	
Detectable (≥40 RNA copies/mL)	5 (19)	3 (21)	2 (15)	
Unknown	5 (19)	2 (14)	3 (23)	
Viral load (>40 RNA copies/mL), [median (IQR)]	13,627 (9633–87,786)	75,571 (8841–100,000)	12,026 (10,425–13,627)	1.0000
Baseline regimen includes [n (%)]				
Boosted PI	12 (44)	12 (86)	0 (0)	
Non-boosted PI	1 (4)	1 (7)	0 (0)	
NRTI	1 (4)	1 (7)	0 (0)	
No baseline treatment	13 (48)	0 (0)	13 (48)	

* *p* value obtained comparing ART-experienced with ART-naïve individuals at baseline; ART, antiretroviral therapy; IQR, interquartile range.

## Data Availability

The datasets generated and analyzed during the current study, including clonal HIV-2 protease sequences, are available in GenBank under accession numbers KT588925-KT589104 and PV797882-PV797921. Additional de-identified clinical and laboratory data that support the findings of this study are available from the corresponding author upon reasonable request, subject to institutional and ethical approvals to protect patient confidentiality.

## Supplementary Materials

The following supporting information can be downloaded at: https://www.mdpi.com/article/10.3390/ijms27125183/s1.

## References

[1] Clavel F., Guetard D., Brun-Vezinet F., Chamaret S., Rey M.A., Santos-Ferreira M.O., Laurent A.G., Dauguet C., Katlama C., Rouzioux C. (1986). Isolation of a new human retrovirus from West African patients with AIDS. Science.

[2] Dwyer-Lindgren L., Cork M.A., Sligar A., Steuben K.M., Wilson K.F., Provost N.R., Mayala B.K., VanderHeide J.D., Collison M.L., Hall J.B. (2019). Mapping HIV prevalence in sub-Saharan Africa between 2000 and 2017. Nature.

[3] Visseaux B., Bertine M., Le Hingrat Q., Ferre V., Charpentier C., Collin F., Damond F., Matheron S., Hue S., Descamps D. (2021). HIV-2 diversity displays two clades within group A with distinct geographical distribution and evolution. Virus Evol..

[4] Faria N.R., Hodges-Mameletzis I., Silva J.C., Rodes B., Erasmus S., Paolucci S., Ruelle J., Pieniazek D., Taveira N., Trevino A. (2012). Phylogeographical footprint of colonial history in the global dispersal of human immunodeficiency virus type 2 group A. J. Gen. Virol..

[5] DGS, INSA (2024). Infeção por VIH em Portugal—2024.

[6] Visseaux B., Damond F., Matheron S., Descamps D., Charpentier C. (2016). Hiv-2 molecular epidemiology. Infect. Genet. Evol..

[7] Soriano V., Gomes P., Heneine W., Holguin A., Doruana M., Antunes R., Mansinho K., Switzer W.M., Araujo C., Shanmugam V. (2000). Human immunodeficiency virus type 2 (HIV-2) in Portugal: Clinical spectrum, circulating subtypes, virus isolation, and plasma viral load. J. Med. Virol..

[8] Popper S.J., Sarr A.D., Travers K.U., Gueye-Ndiaye A., Mboup S., Essex M.E., Kanki P.J. (1999). Lower human immunodeficiency virus (HIV) type 2 viral load reflects the difference in pathogenicity of HIV-1 and HIV-2. J. Infect. Dis..

[9] Ceccarelli G., Giovanetti M., Sagnelli C., Ciccozzi A., d’Ettorre G., Angeletti S., Borsetti A., Ciccozzi M. (2021). Human Immunodeficiency Virus Type 2: The Neglected Threat. Pathogens.

[10] Tchounga B., Ekouevi D.K., Balestre E., Dabis F. (2016). Mortality and survival patterns of people living with HIV-2. Curr. Opin. HIV AIDS.

[11] Esbjornsson J., Mansson F., Kvist A., da Silva Z.J., Andersson S., Fenyo E.M., Isberg P.E., Biague A.J., Lindman J., Palm A.A. (2019). Long-term follow-up of HIV-2-related AIDS and mortality in Guinea-Bissau: A prospective open cohort study. Lancet HIV.

[12] Moranguinho I., Taveira N., Bartolo I. (2023). Antiretroviral Treatment of HIV-2 Infection: Available Drugs, Resistance Pathways, and Promising New Compounds. Int. J. Mol. Sci..

[13] Smith R.A., Raugi D.N., Nixon R.S., Seydi M., Margot N.A., Callebaut C., Gottlieb G.S. (2024). The University of Washington–Senegal HIV-2 Study Group. Antiviral Activity of Lenacapavir Against Human Immunodeficiency Virus Type 2 (HIV-2) Isolates and Drug-Resistant HIV-2 Mutants. J. Infect. Dis..

[14] Neverette N.C., Dumond J.B., McMahon D.K., Devanathan A.S. (2025). Lenacapavir: Playing the Long Game in the New Era of Antiretrovirals. Clin. Pharmacol. Ther..

[15] Smith R.A., Anderson D.J., Pyrak C.L., Preston B.D., Gottlieb G.S. (2009). Antiretroviral drug resistance in HIV-2: Three amino acid changes are sufficient for classwide nucleoside analogue resistance. J. Infect. Dis..

[16] Pacheco P., Marques N., Rodrigues P., Mansinho K., Maltez F., Janeiro N., Franco C., Trigo D., Batista J., Duque L. (2023). Safety and Efficacy of Triple Therapy With Dolutegravir Plus 2 Nucleoside Reverse Transcriptase Inhibitors in Treatment-Naive Human Immunodeficiency Virus Type 2 Patients: Results from a 48-Week Phase 2 Study. Clin. Infect. Dis..

[17] Lessells R.J., Avalos A., de Oliveira T. (2013). Implementing HIV-1 genotypic resistance testing in antiretroviral therapy programs in Africa: Needs, opportunities, and challenges. AIDS Rev..

[18] Jespersen S., Mansson F., Lindman J., Wejse C., Medina C., da Silva Z.J., Te D., Medstrand P., Esbjornsson J., Honge B.L. (2020). HIV treatment in Guinea-Bissau: Room for improvement and time for new treatment options. AIDS Res. Ther..

[19] Raugi D.N., Diallo K., Diallo M.B., Faye D., Cisse O., Smith R.A., Sall F., Sall E.H.I., Faye K., Diatta J.P. (2021). Resource and infrastructure challenges on the RESIST-2 Trial: An implementation study of drug resistance genotype-based algorithmic ART switches in HIV-2-infected adults in Senegal. Trials.

[20] Umumararungu T., Nyandwi J.B., Katandula J., Twizeyimana E., Claude Tomani J., Gahamanyi N., Ishimwe N., Olawode E.O., Habarurema G., Mpenda M. (2024). Current status of the small molecule anti-HIV drugs in the pipeline or recently approved. Bioorg. Med. Chem..

[21] WHO (2021). HIV Drug Resistance Strategy, 2021 Update. https://www.who.int/publications/i/item/9789240030565.

[22] Szojka Z., Karlson S., Jansson M., Medstrand P. (2019). Quantification of HIV-2 DNA in Whole Blood. Bio-protocol.

[23] Raugi D.N., Nixon R.S., Leong S., Faye K., Diatta J.P., Sall F., Smith R.A., Sall E.I., Malomar J.J., Seydi M. (2020). HIV-2 Drug Resistance Genotyping from Dried Blood Spots. J. Clin. Microbiol..

[24] WHO (2020). WHO Manual for HIV Drug Resistance Testing Using Dried Blood Spot Specimens.

[25] Parreira R., Monteiro F., Padua E., Piedade J., Venenno T., Paixao M.T., Esteves A. (2006). Natural polymorphisms of HIV type 2 pol sequences from drug-naive individuals. AIDS Res. Hum. Retroviruses.

[26] Gottlieb G.S., Hawes S.E., Wong K.G., Raugi D.N., Agne H.D., Critchlow C.W., Kiviat N.B., Sow P.S. (2008). HIV type 2 protease, reverse transcriptase, and envelope viral variation in the PBMC and genital tract of ARV-naive women in Senegal. AIDS Res. Hum. Retroviruses.

[27] Raugi D.N., Smith R.A., Ba S., Toure M., Traore F., Sall F., Pan C., Blankenship L., Montano A., Olson J. (2013). Complex patterns of protease inhibitor resistance among antiretroviral treatment-experienced HIV-2 patients from Senegal: Implications for second-line therapy. Antimicrob. Agents Chemother..

[28] Charpentier C., Berzow D., Le Hingrat Q., Kaiser R., Gomes P., Miranda A.C., Damond F., Ghosn J., van Kampen J.J.A., Jensen B.O. (2025). HIV-2EU—Supporting Standardized HIV-2 Drug Resistance Interpretation: An Update. Clin. Infect. Dis..

[29] Chu H., Gange S.J., Yamashita T.E., Hoover D.R., Chmiel J.S., Margolick J.B., Jacobson L.P. (2005). Individual variation in CD4 cell count trajectory among human immunodeficiency virus-infected men and women on long-term highly active antiretroviral therapy: An application using a Bayesian random change-point model. Am. J. Epidemiol..

[30] Wittkop L., Arsandaux J., Trevino A., Schim van der Loeff M., Anderson J., van Sighem A., Boni J., Brun-Vezinet F., Soriano V., Boufassa F. (2017). CD4 cell count response to first-line combination ART in HIV-2+ patients compared with HIV-1+ patients: A multinational, multicohort European study. J. Antimicrob. Chemother..

[31] Tzou P.L., Descamps D., Rhee S.Y., Raugi D.N., Charpentier C., Taveira N., Smith R.A., Soriano V., de Mendoza C., Holmes S.P. (2020). Expanded Spectrum of Antiretroviral-Selected Mutations in Human Immunodeficiency Virus Type 2. J. Infect. Dis..

[32] Storto A., Visseaux B., Bertine M., Le Hingrat Q., Collin G., Damond F., Khuong M.A., Blum L., Tubiana R., Karmochkine M. (2018). Minority resistant variants are also present in HIV-2-infected antiretroviral-naive patients. J. Antimicrob. Chemother..

[33] de Pina-Araujo I.I.M., Guimaraes M.L., Bello G., Vicente A.C., Morgado M.G. (2014). Profile of the HIV epidemic in Cape Verde: Molecular epidemiology and drug resistance mutations among HIV-1 and HIV-2 infected patients from distinct islands of the archipelago. PLoS ONE.

[34] Colson P., Henry M., Tivoli N., Gallais H., Gastaut J.A., Moreau J., Tamalet C. (2005). Polymorphism and drug-selected mutations in the reverse transcriptase gene of HIV-2 from patients living in southeastern France. J. Med. Virol..

[35] Bercoff D.P., Triqueneaux P., Lambert C., Oumar A.A., Ternes A.M., Dao S., Goubau P., Schmit J.C., Ruelle J. (2010). Polymorphisms of HIV-2 integrase and selection of resistance to raltegravir. Retrovirology.

[36] Cavaco-Silva J., Aleixo M.J., Van Laethem K., Faria D., Valadas E., Goncalves Mde F., Gomes P., Vandamme A.M., Cunha C., Camacho R.J. (2013). Mutations selected in HIV-2-infected patients failing a regimen including atazanavir. J. Antimicrob. Chemother..

[37] De La Cruz J., Vardhanbhuti S., Sahoo M.K., Rovner R., Bosch R.J., Manasa J., Katzenstein D.A., Pinsky B.A. (2019). Persistence of Human Immunodeficiency Virus-1 Drug Resistance Mutations in Proviral Deoxyribonucleic Acid After Virologic Failure of Efavirenz-Containing Antiretroviral Regimens. Open Forum Infect. Dis..

[38] Navarrete-Munoz M.A., Ramos R., Holguin A., Cabello A., Gorgolas M., Benito J.M., Rallon N. (2022). High frequency of CD8 escape mutations in elite controllers as new obstacle for HIV cure. Virulence.

[39] Pasternak A.O., Berkhout B. (2023). HIV persistence: Silence or resistance?. Curr. Opin. Virol..

[40] Avila-Rios S., Parkin N., Swanstrom R., Paredes R., Shafer R., Ji H., Kantor R. (2020). Next-Generation Sequencing for HIV Drug Resistance Testing: Laboratory, Clinical, and Implementation Considerations. Viruses.

[41] Balakrishna S., Loosli T., Zaheri M., Frischknecht P., Huber M., Kusejko K., Yerly S., Leuzinger K., Perreau M., Ramette A. (2023). Frequency matters: Comparison of drug resistance mutation detection by Sanger and next-generation sequencing in HIV-1. J. Antimicrob. Chemother..

[42] Metzner K.J. (2022). Technologies for HIV-1 drug resistance testing: Inventory and needs. Curr. Opin. HIV AIDS.

[43] Lee E.R., Parkin N., Jennings C., Brumme C.J., Enns E., Casadella M., Howison M., Coetzer M., Avila-Rios S., Capina R. (2020). Performance comparison of next generation sequencing analysis pipelines for HIV-1 drug resistance testing. Sci. Rep..

[44] Goncalves F., Cabanas J., Costa I., Veloso M., Ribeiro M., Fernandes S., Diogo I., Sebastiao C.S., Pingarilho M., Pimentel V. (2025). Hybrid next-generation sequencing protocol for testing HIV-2 drug resistance. J. Virol. Methods.

[45] Ouyang F., Yuan D., Zhai W., Liu S., Zhou Y., Yang H. (2024). HIV-1 Drug Resistance Detected by Next-Generation Sequencing among ART-Naive Individuals: A Systematic Review and Meta-Analysis. Viruses.

[46] Kuniholm J., Coote C., Henderson A.J. (2022). Defective HIV-1 genomes and their potential impact on HIV pathogenesis. Retrovirology.

[47] Quan Y., Xu H., Wainberg M.A. (2014). Defective HIV-1 quasispecies in the form of multiply drug-resistant proviral DNA within cells can be rescued by superinfection with different subtype variants of HIV-1 and by HIV-2 and SIV. J. Antimicrob. Chemother..

[48] Melandri M., Faua C., Gantner P. (2026). The HIV-2 reservoir: Lessons from HIV-1 and knowledge gaps. Infect. Dis. Now..

[49] Chu C., Armenia D., Walworth C., Santoro M.M., Shafer R.W. (2022). Genotypic Resistance Testing of HIV-1 DNA in Peripheral Blood Mononuclear Cells. Clin. Microbiol. Rev..

[50] Liang B., Luo M., Scott-Herridge J., Semeniuk C., Mendoza M., Capina R., Sheardown B., Ji H., Kimani J., Ball B.T. (2011). A comparison of parallel pyrosequencing and sanger clone-based sequencing and its impact on the characterization of the genetic diversity of HIV-1. PLoS ONE.

[51] Hsieh A.Y.Y., Hassan A.S., Nazziwa J., Lindquist L., Karlson S., Hare J., Kamali A., Karita E., Kilembe W., Price M.A. (2026). Single genome amplification and molecular cloning of HIV-1 populations in acute HIV-1 infection: Implications for studies on HIV-1 diversity and evolutionary rate. Virus Evol..

[52] Auld A.F., Ekra K.A., Shiraishi R.W., Tuho M.Z., Kouakou J.S., Mohamed F., Ettiegne-Traore V., Sabatier J., Essombo J., Adjorlolo-Johnson G. (2014). Temporal trends in treatment outcomes for HIV-1 and HIV-2-infected adults enrolled in Cote d’Ivoire’s national antiretroviral therapy program. PLoS ONE.

[53] Larkin M.A., Blackshields G., Brown N.P., Chenna R., McGettigan P.A., McWilliam H., Valentin F., Wallace I.M., Wilm A., Lopez R. (2007). Clustal W and Clustal X version 2.0. Bioinformatics.

[54] Nicholas H.B., Deerfield D.W.I. (1997). GeneDoc: Analysis and visualization of genetic variation. Embnet News.

[55] Lapp Z., Yoon H., Foley B., Leitner T. (2025). Hypermut 3: Identifying specific mutational patterns in a defined nucleotide context that allows multistate characters. Bioinform. Adv..

[56] Nguyen L.T., Schmidt H.A., von Haeseler A., Minh B.Q. (2015). IQ-TREE: A fast and effective stochastic algorithm for estimating maximum-likelihood phylogenies. Mol. Biol. Evol..

[57] Kalyaanamoorthy S., Minh B.Q., Wong T.K.F., von Haeseler A., Jermiin L.S. (2017). ModelFinder: Fast model selection for accurate phylogenetic estimates. Nat. Methods.

[58] Letunic I., Bork P. (2021). Interactive Tree Of Life (iTOL) v5: An online tool for phylogenetic tree display and annotation. Nucleic Acids Res..

[59] Benjamini Y., Hochberg Y. (1995). Controlling the false discovery rate: A practical and powerful approach to multiple testing. J. R. Stat. Soc. Ser. B (Methodol.).

[60] R Core Team (2026). R: A Language and Environment for Statistical Computing.

[61] Bates D., Maechler M., Bolker B., Walker S. (2015). Fitting Linear Mixed-Effects Models Using lme4. J. Stat. Softw..

[62] Kuznetsova A., Brockhoff P.B., Christensen R.H.B. (2017). lmerTest Package: Tests in Linear Mixed Effects Models. J. Stat. Softw..

[63] Pinheiro J., Bates D., R Core Team (2026). nlme: Linear and Nonlinear Mixed Effects Models. https://CRAN.R-project.org/package=nlme.

[64] Pinheiro J.C., Bates D.M. (2000). Mixed-Effects Models in S and S-PLUS.

