# Supplementary material for: Longitudinal Analysis of HIV-2 Proviral DNA Reveals Archived Protease Inhibitor Resistance and Reservoir Evolution over Eight Years
Source: Int J Mol Sci. 2026 Jun 8;27(12):5183. doi: 10.3390/ijms27125183 (PMC13300208; doi:10.3390/ijms27125183)
Supplement: Supplementary file 1 [file ijms-27-05183-s001.zip › ijms-4291011-supplementary.pdf]

Supplementary data

Table S1. Trend in CD4$^+$ T cell count and viral load during follow-up.

Parameters	All participants								
	**Baseline**	**Year 1**	**Year 2**	**Year 3**	**Year 4**	**Year 5**	**Year 6**	**Year 7**	**Year 8**
**Number**	27	25	25	23	23	22	21	18	17
**CD4$^+$ T-cell count (cell/µl) [Median (IQR)]**	448 (254-600)	458 (315-681)	540 (347-795)	564 (393-860)	620 (386-837)	546 (315-755)	504 (343-823)	666 (370-881)	507 (399-840)
**CD4$^+$ T-cell count category [n (%)]**									
<200 cells/µl	6 (22)	5 (20)	2 (8)	1 (4)	0 (0)	1 (5)	1 (5)	1 (6)	1 (6)
≥200 cells/µl	21 (78)	17 (68)	19 (76)	19 (83)	20 (87)	19 (86)	19 (90)	16 (89)	16 (94)
Unknown	0 (0)	3 (12)	4 (16)	3 (13)	3 (13)	2 (9)	1 (5)	1 (6)	0 (0)
**Viral load, category [n (%)]**									
Undetectable (<40 RNA copies/ml)	17 (62)	13 (52)	11 (44)	13 (57)	12 (52)	14 (64)	12 (57)	8 (44)	10 (59)
Detectable (>40 RNA copies/ml)	5 (19)	5 (20)	5 (20)	3 (13)	4 (18)	3 (14)	4 (19)	2 (12)	4 (24)
Unknown	5 (19)	7 (28)	9 (36)	7 (30)	7 (30)	5 (23)	5 (24)	8 (44)	3 (18)

IQR, interquartile range

Table S2. Trend in CD4+ T cell count and viral load during follow-up in the ART-experienced participants.

Parameters	ART-experienced participants								
	Baseline	Year 1	Year 2	Year 3	Year 4	Year 5	Year 6	Year 7	Year 8
**Number**	14	15	15	13	14	14	14	13	16
**$CD4^+$ T cell count (cell/µl) [Median (IQR)]**	259 (144-600)	398 (126-656)	429 (217-759)	536 (277-636)	564 (346-828)	500 (297-633)	499 (261-790)	671 (361-900)	631 (392-853)
**$CD4^+$ T cell count category [n (%)]**									
<200 cells/µl	6 (43)	5 (33)	2 (13)	1 (8)	0 (0)	1 (7)	1 (7)	1 (8)	1 (6)
≥200 cells/µl	8 (57)	9 (60)	12 (80)	10 (77)	14 (100)	13 (93)	13 (93)	11 (85)	15 (94)
Unknown	0 (0)	1 (7)	1 (7)	2 (15)	0 (0)	0 (0)	0 (0)	1 (8)	0 (0)
**Viral load (RNA copies/ml), category [n (%)]**									
Undetectable (<40 RNA copies/ml)	9 (64)	8 (53)	7 (47)	7 (54)	8 (57)	8 (57)	6 (43)	5 (38)	9 (56)
Detectable (≥40 RNA copies/ml) [n (%)]	3 (21)	4 (27)	3 (20)	2 (15)	3 (21)	3 (21)	4 (29)	2 (15)	4 (25)
Unknown	2 (14)	3 (20)	5 (33)	4 (31)	3 (21)	3 (21)	4 (29)	6 (46)	3 (19)

IQR, interquartile range

Table S3. Trend in CD4+ T cell count and viral load during follow-up in the ART-naïve participants.

Parameters	ART-naïve participants								
	**Baseline**	**Year 1**	**Year 2**	**Year 3**	**Year 4**	**Year 5**	**Year 6**	**Year 7**	**Year 8**
**Number**	13	10	10	10	9	8	7	5	1
**$CD4^+$ T cell count (cell/µl) [Median (IQR)]**	557 (456-1192)	584 (444-1153)	716 (540-985)	638 (440-1299)	671 (422-1557)	773 (423-1400)	792 (389-1311)	557 (425-1269)	433
**$CD4^+$ T cell count category [n (%)]**									
<200 cells/µl	0 (0)	0 (0)	0 (0)	0 (0)	0(0)	0 (0)	0 (0)	0 (0)	0 (0)
≥200 cells/µl	13 (100)	8 (80)	7 (70)	9 (90)	6 (67)	6 (75)	6 (86)	5 (100)	1 (100)
Unknown	0 (0)	2 (20)	3 (30)	1 (10)	3 (33)	2 (25)	1 (14)	0 (0)	0 (0)
**Viral load (RNA copies/ml), category [n (%)]**									
Undetectable (<40 RNA copies/ml)	8 (62)	5 (50)	4 (40)	6 (60)	4 (44)	6 (75)	6 (86)	3 (60)	1 (100)
Detectable (≥40 RNA copies/ml) [n (%)]	2 (15)	1 (10)	2 (20)	1 (10)	1 (11)	0 (0)	0 (0)	0 (0)	0 (0)
Unknown	3 (23)	4 (40)	4 (40)	3 (30)	4 (44)	2 (25)	1 (14)	2 (40)	0 (0)

Table S4. Shannon entropy analysis between sequences from ART- naïve and ART-experienced participants at baseline.

Position	Query consensus	Entropy difference between background and query (Hdiff)	# randomized entropy diff>=Hdiff	Highest randomized entropy diff	P-value
1	P	0.040	7250	-0.312	0.73
2	Q	0.000	10000	0.000	1.00
3	F	0.000	10000	0.000	1.00
4	S	0.000	10000	0.000	1.00
5	L	0.000	10000	0.000	1.00
6	W	0.000	10000	0.000	1.00
7	K	0.304	348	0.544	0.03
8	R	0.018	8381	0.392	0.84
9	P	-0.002	8005	0.300	0.80
10	V	-0.047	5884	0.257	0.59
11	V	0.057	4901	0.202	0.49
12	T	0.114	1495	-0.280	0.15
13	A	0.000	10000	0.000	1.00
14	H	0.130	780	-0.264	0.08
15	I	0.614	0	-0.417	≤0.0005
16	E	0.054	5829	-0.312	0.58
17	G	0.051	6433	0.429	0.64
18	Q	0.000	10000	0.000	1.00
19	P	-0.060	5855	-0.419	0.59
20	V	0.000	10000	0.000	1.00
21	E	0.372	2	0.391	≤0.0005
22	V	-0.104	1392	-0.239	0.14

23	L	0.057	4815	-0.209	0.48
24	L	0.000	10000	0.000	1.00
25	D	0.114	1361	0.269	0.14
26	T	-0.062	4109	0.337	0.41
27	G	-0.163	581	-0.318	0.06
28	A	0.057	4929	-0.209	0.49
29	D	0.156	765	-0.342	0.08
30	D	0.097	3576	0.370	0.36
31	S	0.057	4881	-0.209	0.49
32	I	0.054	6386	-0.320	0.64
33	V	0.057	4901	0.202	0.49
34	A	0.000	10000	0.000	1.00
35	G	-0.059	2886	-0.177	0.29
36	I	-0.059	2981	-0.239	0.30
37	E	-0.304	59	0.404	0.01
38	L	0.000	10000	0.000	1.00
39	G	-0.181	585	0.351	0.06
40	S	-0.547	0	-0.494	≤0.0005
41	N	0.139	2425	0.476	0.24
42	Y	-0.002	8684	0.306	0.87
43	S	-0.094	3884	0.447	0.39
44	P	0.000	10000	0.000	1.00
45	K	0.057	4832	0.230	0.48
46	I	-0.405	0	0.391	≤0.0005
47	V	0.000	10000	0.000	1.00
48	G	-0.059	2947	-0.209	0.29
49	G	0.000	10000	0.000	1.00
50	I	-0.059	2957	0.230	0.30

51	G	0.114	1416	0.329	0.14
52	G	0.000	10000	0.000	1.00
53	F	0.000	10000	0.000	1.00
54	I	-0.143	625	-0.267	0.06
55	N	-0.086	3784	-0.350	0.38
56	T	0.170	543	-0.343	0.05
57	K	-0.239	74	0.307	0.01
58	E	-0.104	1372	-0.239	0.14
59	Y	0.057	4845	0.202	0.48
60	K	0.442	11	-0.516	0.0001
61	N	0.359	98	0.560	0.01
62	V	0.000	10000	0.000	1.00
63	E	0.000	10000	0.000	1.00
64	I	-0.400	3	-0.424	≤0.0005
65	E	-0.022	7895	-0.334	0.79
66	V	0.057	4876	-0.209	0.49
67	L	0.057	4845	0.202	0.48
68	N	0.141	1438	-0.358	0.14
69	K	0.100	2051	0.230	0.21
70	K	-0.236	73	0.336	0.01
71	V	-0.013	8791	0.402	0.88
72	R	-0.410	1	-0.508	≤0.0005
73	A	-0.086	3798	-0.353	0.38
74	T	0.000	10000	0.000	1.00
75	I	-0.293	12	-0.405	0.0001
76	M	-0.165	1452	-0.398	0.15
77	T	0.307	5	-0.318	≤0.0005
78	G	0.000	10000	0.000	1.00

79	D	0.270	112	-0.443	0.01
80	T	-0.059	3001	-0.209	0.30
81	P	0.000	10000	0.000	1.00
82	I	-0.143	625	-0.267	0.06
83	N	-0.002	7946	-0.312	0.79
84	I	-0.136	2633	-0.485	0.26
85	F	0.000	10000	0.000	1.00
86	G	0.000	10000	0.000	1.00
87	R	0.111	2392	-0.359	0.24
88	N	0.114	1396	-0.284	0.14
89	I	0.121	1642	-0.364	0.16
90	L	-0.183	598	-0.434	0.06
91	T	0.126	1785	-0.363	0.18
92	A	-0.035	7370	-0.397	0.74
93	L	-0.029	7869	0.370	0.79
94	G	0.000	10000	0.000	1.00
95	M	0.000	10000	0.000	1.00
96	S	0.057	5000	-0.209	0.50
97	L	0.000	10000	0.000	1.00
98	N	0.114	1335	-0.246	0.13
99	L	-0.363	3	-0.442	≤0.0005

Table S5. Shannon entropy between sequences of participants who had experienced ART at baseline and year 8 of the study.

Position	Query consensus	Entropy difference between background and query (Hdiff)	# randomized entropy diff>=Hdiff	Highest randomized entropy diff	P-value
1	P	0.059	5968	-0.369	0.60
2	Q	0.000	10000	0.000	1.00
3	F	0.000	10000	0.000	1.00
4	S	0.000	10000	0.000	1.00
5	L	0.000	10000	0.000	1.00
6	W	0.000	10000	0.000	1.00
7	K	0.163	2375	-0.501	0.24
8	R	0.119	3394	-0.501	0.34
9	P	0.059	5976	-0.305	0.60
10	V	-0.265	509	-0.457	0.05
11	V	0.000	10000	0.000	1.00
12	T	0.000	10000	0.000	1.00
13	A	0.000	10000	0.000	1.00
14	H	-0.001	9005	0.214	0.90
15	I	-0.310	82	-0.425	0.01
16	E	-0.076	5262	-0.527	0.53
17	G	0.143	2561	-0.425	0.26
18	Q	0.000	10000	0.000	1.00
19	P	0.013	9271	0.491	0.93
20	V	-0.136	1487	-0.369	0.15
21	E	-0.363	48	-0.527	0.0001
22	V	0.104	4167	-0.369	0.42
23	L	0.000	10000	0.000	1.00

24	L	0.000	10000	0.000	1.00
25	D	0.000	10000	0.000	1.00
26	T	0.119	3317	-0.453	0.33
27	G	0.163	2420	-0.497	0.24
28	A	0.000	10000	0.000	1.00
29	D	0.000	10000	0.000	1.00
30	D	0.059	6000	-0.305	0.60
31	S	0.000	10000	0.000	1.00
32	I	0.059	5962	-0.369	0.60
33	V	-0.136	1514	-0.425	0.15
34	A	0.000	10000	0.000	1.00
35	G	-0.076	5244	-0.369	0.52
36	I	0.059	5893	-0.305	0.59
37	E	0.304	703	-0.658	0.07
38	L	0.000	10000	0.000	1.00
39	G	0.089	5450	0.476	0.55
40	S	0.192	2277	0.719	0.23
41	N	-0.054	6953	0.590	0.70
42	Y	0.059	6035	-0.369	0.60
43	S	-0.098	5173	0.575	0.52
44	P	0.000	10000	0.000	1.00
45	K	0.000	10000	0.000	1.00
46	I	0.269	496	0.521	0.05
47	V	-0.136	1421	-0.305	0.14
48	G	0.059	6000	-0.305	0.60
49	G	0.000	10000	0.000	1.00
50	I	0.059	6041	-0.305	0.60
51	G	0.000	10000	0.000	1.00

52	G	0.000	10000	0.000	1.00
53	F	0.000	10000	0.000	1.00
54	I	-0.162	2497	-0.517	0.25
55	N	0.143	2607	-0.369	0.26
56	T	0.000	10000	0.000	1.00
57	K	0.239	790	-0.413	0.08
58	E	0.104	4124	-0.369	0.41
59	Y	0.000	10000	0.000	1.00
60	K	0.059	6057	-0.305	0.61
61	N	0.013	9218	0.476	0.92
62	V	-0.136	1474	-0.369	0.15
63	E	0.000	10000	0.000	1.00
64	I	0.400	115	-0.590	0.01
65	E	0.121	2168	0.421	0.22
66	V	0.000	10000	0.000	1.00
67	L	0.000	10000	0.000	1.00
68	N	-0.183	1003	0.479	0.10
69	K	-0.229	294	-0.369	0.03
70	R	-0.135	2485	0.565	0.25
71	V	0.156	2706	0.491	0.27
72	R	0.410	155	0.618	0.02
73	A	0.143	2592	-0.425	0.26
74	T	0.000	10000	0.000	1.00
75	I	0.157	2891	-0.474	0.29
76	M	0.222	1352	-0.569	0.14
77	T	0.000	10000	0.000	1.00
78	G	0.000	10000	0.000	1.00
79	D	-0.076	5288	-0.541	0.53

80	T	0.059	6057	-0.305	0.61
81	P	0.000	10000	0.000	1.00
82	I	-0.162	2463	-0.474	0.25
83	N	0.059	6015	-0.369	0.60
84	I	0.001	9951	0.703	1.00
85	F	-0.136	1477	-0.310	0.15
86	G	0.000	10000	0.000	1.00
87	R	0.059	6028	-0.369	0.60
88	N	0.000	10000	0.000	1.00
89	I	-0.132	3051	0.559	0.31
90	L	0.156	2716	0.521	0.27
91	T	-0.201	1443	-0.425	0.14
92	A	0.341	165	0.521	0.02
93	L	0.143	2553	-0.370	0.26
94	G	0.000	10000	0.000	1.00
95	M	0.000	10000	0.000	1.00
96	S	0.000	10000	0.000	1.00
97	L	0.000	10000	0.000	1.00
98	N	0.000	10000	0.000	1.00
99	L	0.363	117	0.547	0.01

Table S6. Drug resistance mutations, and natural polymorphisms in clonal PR sequences obtained from the samples collected at baseline

Participant ID	Clone number	Major mutation	Accessory mutation	Polymorphism	ART
PTHSM1	23	I84V	-	Y14H, N40S, E65K, K70T	AZT + 3TC + LPV/r
	32	-	-	Y14H, N40S, E65K, K70T	
	33	-	-	R8S, Y14H, N40S, E65K, K70T	
	40	-	-	Y14H, E16G, E65K, K70T, R72Q	
	41	-	-	Y14H, N40S, E65K, K70T	
	42	-	-	Y14H, N40S, E65K, K70T	
PTHSM2	1	-	-	N40S, K70R	3TC + AZT + SQV /r
	2	-	-	N40S, K70R	
	4	-	-	N40S	
	6	-	-	E37G, N40S, I64M, K70R	
	10	-	-	N40S	
	11	-	-	T26M, N40S, K70R	
	12	-	-	Y14H, N40S, K70R	
	13	-	-	N40S, K70G, T80I	
PTHSM3	1	I84V, L90M	I64V, V71I, L99F	N40G, I46V, E65K, A92T, L93I	TDF + RAL + DRV/r
	2	I84V, L90M	I64V, V71I, L99F	N40G, I46V, E65K, I75V, A92T	
	3	I84V, L90M	I64V, V71I, L99F	N40G, I46V, E65K, I75V, A92T	
	4	I84V, L90M	I64V, V71I, L99F	N40G, I46V, E65K, I75V, A92T	
	5	I84V, L90M	I64V, V71I, L99F	N40G, I46V, E65K, I75V, A92T	
	6	I84V, L90M	I64V, V71I, L99F	N40G, I46V, E65K, I75V, A92T, L93I	
	7	I84V, L90M	I64V, V71I, L99F	R8G, N40G, I46V, E65K, I75V, A92T	
	8	I84V, L90M	I64V, V71I, L99F	I32M, N40G, E65K, A92T, L93I	
	9	I84V, L90M	I64V, V71I, L99F	N40G, I46V, E65K, I75V, A92T	

	10	I84V, L90M	I64V, V71I, L99F	N40G, I46V, E65K, I75V, A92T	
**PTHSM4**	1	I54M, I82F, L90M	V71I, A73G	K7R, V22I, E37D, N40S, N68G, K70R, M76L, T91A	AZT + 3TC + LPV/r
	3	I54M, I82F, L90M	V71I, A73G	K7R, V22I, E37D, N40S, N68G, K70R, M76L, T91A	
**PTHSM5**	1	-	-	Y14H, N68G	AZT + 3TC + LPV/r
	10	-	-	Y14H, N40S, N68G	
	12	-	-	Y14H, N40S, K70R, I89V	
	13	-	-	Y14H, N40S, K70R, I89V	
	14	-	-	Y14H, N40S, I89V	
	15	-	-	Y14H, N40S, N68G, I89V	
	2	-	-	Y14H, D30G, N40S, G48R, N68G, K70R, I89V	
	5	-	-	Y14H, E37G, N40S, N68G, I89V	
	9	-	-	Y14H, N40S, N68G	
**PTHSM6**	9	-	-	G17D, N40S, S43R, E65K	AZT + 3TC + SQV/r
	26	-	-	P1T, N40S, E65K	
	28	-	-	N40S, E65K	
	29	-	-	G17D, E37K, E65K	
	32	-	-	N40S, E65K	
	33	-	-	K7T, G17D, N40S, E65K	
**PTHSM7**	1	-	-	Y14H, G27R, N40S, N68G, R72K	AZT + 3TC + LPV/r
	3	-	-	Y14H, N40S, N68G, R72K	
	6	-	-	Y14H, N40S, Y42C, N55D, N68G, R72K	

PTHSM8	2	-	-	Y14H, N40S, E65R	AZT + 3TC + IDV/r
	4	-	-	Y14H, N40S, K60N, E65R, M76V	
	8	-	-	Y14H, I15V, N40S, E65R	
	11	-	-	Y14H, N40S, E65R	
	12	-	-	Y14H, E37G, N40S, E65R, R72G	
	19	-	-	Y14H, N40S, E65R	
PTHSM9	1	-	L99F	P19S, G39R, N61D, E65K	AZT + 3TC
	6	-	-	P19S, G39R, N61D, E65K	
	7	-	-	P19S, G39R, N61D, E65K	
	8	-	-	P19S, G39R, N61D, E65K	
	10	-	-	P9LP, P19S, G39R, N61D, E65K	
	11	-	-	P19S, G39R, N61D, E65K	
	14	-	-	P19S, G39R, N61D, E65K	
	15	-	-	P19S, G39R, N61D, E65K	
	16	-	-	P19S, G39R, I50T, N61D, E65K	
PTHSM10	7	-	-	Y14H, I36L, I46V, K57R, N68G, K70R	TDF + FTC + LPV/r
	10	-	-	Y14H, N40S, I46V, K57R, N68G, K70R	
	13	-	-	Y14H, K57R, E58V, N68G, K70R	
	16	-	-	Y14H, I46V, K57R, N68G, K70R, M76T	
	17	-	-	Y14H, K57R, E58V, N68G, K70R	
	18	-	-	Y14H, I46V, K57R, N68G, K70R, D79G	
PTHSM11	6	-	V10I	N40S, K70R, I89V	TDF + FTC + LPV/r
	22	I54M, I82F	V10I	K70R, I89V	
PTHSM12	1	-	-	Y14H, N40S, N41D, S43T, E65R, K70R	ABC + 3TC + SQV/r
	2	-	-	Y14H, N40S, N41D, S43T, E65R, K70R	

	11	-	-	Y14H, N40S, N41D, S43T, E65R, K70R	
	13	-	-	Y14H, N40S, N41D, S43T, E65R, K70R	
	14	-	-	Y14H, T26A N40S, N41D, S43T, E65R, K70R	
	16	-	-	Y14H, N40S, N41D, S43T, E65R, K70R	
	18	-	-	Y14H, N41D, S43T, E65R, K70R	
	19	-	-	Y14H, N41D, S43T, E65R, K70R	
	20	-	-	Y14H, N40S, N41D, S43T, E65R, K70R	
**PTHSM13**	1	-	-	N40S, N68G	AZT + 3TC + LPV/r
	2	-	-	G27E, N40S, N55D, N68G	
	3	-	-	G27E, N40S, N55D, N68G	
	8	-	-	Y14H, N40S, N68G, K70R	
	10	-	-	Y14H, N40S, N68G, N83D	
	12	-	-	N40S, N68G, R87K	
**PTHSM27**	69	-	-	Y14H, N40C, N41D, N68G, K70R, R72K, I89V	ABC + 3TC + SQV/r
	68	-	A73G	Y14H, N40C, N68G, K70R, I89V	
	65	-	-	Y14H, N40S, N41H, N68G, K70R, R72K	
	64	-	-	Y14H, N40C, N41D, N68G, K70R, R72K, I89V	
	51	-	-	Y14H, N40C, N41D, N68G, K70R, R72K, I89V	
	49	-	-	Y14H, N40C, N41D, N68G, K70R, R72K, I89V	
	47	-	-	Y14H, N40C, N41D, N68G, K70R, R72K, I89V	
**PTHSM14**	2	-	-	E21D, N40S, N61D, E65K	No treatment
	4	-	-	I15M, E21D, N40S, N61D, E65K	

	17	-	-	E21D, N40S, N61D, E65K	
	31	-	-	T12R, E21D, N40S, N61D, E65K	
	33	-	-	E21D, N40S, N61D, E65K	
	34	L90M	-	E21D, N40S, S43T, N61D, E65K, I84L, L93I	
	35	L90M	V71I	E21D, L23S, N40S, S43T, K60H, E65K, K69R, D79E, I84L	
	36	-	-	Y14H, E21D, N40S, S43T, N61D, E65K	
	37	-	V10I	E21D, D25V, N40S, N61D, E65K	
	40	L90M	V71I	E21D, N40S, S43T, K60H, E65K, K69R, D79E, I84L	
	43	L90M	V71I	E21D, N40S, N61D, E65K, I89V	
**PTHSM15**	1	-	-	N40S, N68G	No treatment
	3	-	-	I15V, N40S, N68G	
	4	-	-	I15V, N40S, N68G	
	9	-	-	I15V, N40S, N68G	
	10	-	-	I15V, N40S, N68G	
	11	-	-	I15V, N40S, N68G	
	12	-	-	I15V, N40S, N68G	
	14	-	-	I15V, N40S, N68G	
	15	-	-	I15V, D25G, N40S, N68G	
	16	-	-	I15V, N40S, N68G	
**PTHSM16**	6	-	-	I15V, N40S, K70R	No treatment
**PTHSM17**	1	-	-	I15V, N68G	No treatment
	2	-	-	Y14H, T26A, D30G	
	4	-	-	Y14H, N68S	
	5	-	-	Y14H, E16G, N68S	
	6	-	-	Y14H, N68S	

	7	-	-	Y14H, N68S	
	8	-	-	K7R, Y14H, N68S	
	9	-	-	Y14H, N68S	
	10	-	-	Y14H, D30G, N68S	
**PTHSM18**	10	-	-	I15V, N40S, K70R	No treatment
	31	-	-	I15V, N40S, K70R	
	35	-	-	I15V, N40S, K70R	
**PTHSM19**	1	-	-	Y14H, N40S, N68G, K70R, R87S, I89V	No treatment
	2	-	-	Y14H, N40S, N68G, K70R, I89V	
	3	-	-	Y14H, N40S, K60R, N68G, K70R, N83S, R87S, I89V	
	5	-	-	Y14H, N40S, N68G, K70R, I89V	
	6	-	-	Y14H, N40S, N68G, K70R, R87S, I89V	
	7	-	-	Y14H, N40S, K60R, N68G, K70R, I89V	
	9	-	-	Y14H, N40S, N68G, K70R, I89V	
	10	-	-	Y14H, N40S, N68G, K70R, I89V	
	11	-	-	Y14H, N40S, N68G, K70R, R87S, I89V	
**PTHSM20**	11	-	-	Y14H, N40S, E65R, D79E	No treatment
	12	-	-	Y14H, S31P, N40S, E65R, D79E	
	13	-	-	Y14H, N40S, E65R, D79E	
	14	-	-	Y14H, N40S, E65R, D79E	
	15	-	-	N40S, E65R, D79E, N88S	
	18	-	-	N40S, E65R, D79E	
	19	-	-	N40S, E65R, D79E, S96T	
	22	-	-	Y14H, I15M, N40S, E65R	
	24	-	-	P1R, Y14H, N40S, Y59C, E65G, L67I, A73V, D79E	
**PTHSM21**	1	-	-	Y14H, N40S, E65K, K70R, I89V	No treatment

	2	-	-	Y14H, N40S, E65K, K70R, I89V	
	4	-	-	Y14H, N40S, E65K, K70R, I89V	
	11	-	-	Y14H, N40S, E65K, K70R, I89V	
	12	-	-	Y14H, N40S, E65K, K70R, I89V	
	13	-	N88D	Y14H, N40S, E65K, K70R, I89V	
	15	-	-	V11A, Y14H, N40S, E65K, K70R, I89V	
	16	-	-	Y14H, N40S, E65K, K70R, I89V	
	17	-	-	Y14H, N40S, E65K, K70R, I89V	
	28	-	-	Y14H, N40S, E65K, K70R, I89V	
**PTHSM22**	11	-	V71I	K7N, Y14H, N40S, N41D, K60Q, E65K, K70R, T77V, A92T	No treatment
	12	-	V71I	K7N, Y14H, N40S, N41D, K60Q, E65K, K70R, T77V, A92T	
	14	-	V71I	K7N, Y14H, G17V, D29V, D30V, N40S, N41D, K60Q, E65K, K70R, T77V, A92T	
	15	-	V71I	K7N, Y14H, N40S, N41D, K60Q, E65K, K70R, T77V, A92T	
	16	-	V71I	K7T, R8S, P9Q, Y14H, E16A, G17V, D29V, N40S, N41A, Y42D, N55K, T56G, K60Q, E65K, K70R, T77V, A92T	
	17	-	V71I	P1R, K7T, R8S, Y14H, G17V, D29A, N40S, N41D, K60Q, E65K, K70R, T77V, A92T	
	18	-	V71I	K7N, Y14H, N40S, N41D, K60Q, E65K, V66A, K70R, T77V, A92T	
	19	-	V71I	K7N, Y14H, G39R, N40S, N41D, G51K, K60Q, E65K, K70R, M76I, T77V, A92T	
	20	-	V71I	K7N, Y14H, N40S, N41D, K60Q, E65K, K70R, T77V, A92T	
**PTHSM23**	2	-	-	I15M, Y14H, N40S, S43T, N61S, E65K, K70R	No treatment
	5	-	-	Y14H, N40S, S43T, N61G, E65K, K70R	
	6	-	-	Y14H, N40S, S43T, N61G, E65K, K70R	

	7	-	-	Y14H, N40S, N61S, E65K, K70R	
	8	-	-	Y14H, N40S, N61S, E65K, K70R	
	9	-	-	Y14H, N40S, N61S, E65K, K70R	
	11	-	-	Y14H, N40S, K45E, N61S, E65K, K70R	
	13	-	-	Y14H, N40S, N61S, E65K, K70R	
	14	-	-	Y14H, N40S, N61S, E65K, K70R	
	15	I84V	-	Y14H, N40S, N61S, E65K, K70R	
**PTHSM24**	2	-	-	G17D, N40S, N41D, T56I, E65K	No treatment
	6	-	-	N40S, N41D, E65K	
	7	-	-	I32V, N40S, N41D, E65K, N98D	
**PTHSM25**	4	L90M	-	I15V, N40S, N41D, E65K	No treatment
	6	-	-	I15V, N40S, N41D, E65K, N98T	
	9	-	-	I15V, N40S, N41D, E65K	
	11	-	-	I15V, N40S, N41D, E65K	
	12	-	-	I15V, N40S, N41D, E65K	
	13	-	-	I15V, N40S, N41D, E65K	
	14	-	-	I15V, N40S, N41D, E65K	
	15	-	-	I15V, N40S, N41D, E65K	
**PTHSM26**	1	-	-	Y14N, I15V, P19S, N40C, E65K, T91A	No treatment
	2	-	-	P19S, I32T, G39R, N40C, T91A	
	3	-	-	P19S, N40C, T91A, L93F	
	6	-	-	R8S, P19S, T21P, E21D, D30V, V33A, G39R, N40C, N41T, T56A, T91A	
	7	-	-	K7E, P19A, N40C, G51E, T91A	
	8	-	-	Y14H, I15V, P19S, N40C, T91A	

Table S7. Drug resistance mutations and natural polymorphisms in clonal PR sequences obtained from the samples collected in final year of the study (year 8).

Participant ID	Clone number	Major mutation	Accessory mutation	Polymorphism
**PTHSM1**	1	-	-	Y14H, V62I, E65K, K70T
	2	-	-	Y14H, G35E, N40S, E65K, K70T
	3	-	-	Y14H, N40S, E65K, K70T
	4	-	-	Y14H, E65K, K70T
	5	-	-	Y14H, N40S, E65K, K70T
	6	-	-	Y14H, E65K, K70T
	7	-	-	Y14H, N40S, E65K, K70T
**PTHSM6**	1	-	-	V20I, N40S, N41D, E65K, D79E
**PTHSM11**	1	I54M, I82F, L90M	V10I, F85L	N40S, S43T, I46V, I84L, I89V
	2	I54M, I82F, L90M	V10I, V33I, V71I	N40G, S43T, I84L
	3	V47A, I82F	V10I	E16K, N40S, S43T, K70R, I89V
**PTHSM12**	1	I54M, I84V	V71I	Y14H, E21Q, N40S, N41D, S43T, E65K, K70R, I75V
	2	-	-	Y14H, N40S, N41D, S43T, E65R, K70R
	3	-	-	Y14H, N40S, N41D, S43T, E65R, K70R
**PTHSM13**	1	-	-	Y14H, N40S, N68G, K70R, I89V
	2	-	-	Y14H, N40S, N68G, K70R
	3	-	-	G39R, N40S, N68G, K70R
	4	-	-	N40S, N68S
	5	-	-	G39R, N40S, N68G, K70R
	6	-	-	N60S, N68G, K70R

	7	-	-	Y14H, N40S, N68G, K70R
	8	-	-	N40S, N68S
	9	-	-	Y14H, N40S, N68G, K70R, I89V
**PTHSM14[a]**	1	-	-	E21D, N40S, N61D, E65K
	2	-	-	E21D, N40S, N61D, E65K
	3	-	-	N40S, N61D, E65K
**PTHSM18[b]**	2	-	V10I	I15V, N40S, K70R
**PTHSM26[c]**	1	-	-	I15V, P19S, N40C, T91A
	2	-	-	I15V, P19S, N40C, T91A
	3	-	-	I15V, P19S, N40C, T91A
**PTHSM27**	1	-	-	Y14H, N40C, N41D, N68G, K70R, I89V
	2	-	-	Y14H, N40C, N41D, N68G, K70R, I89V
	3	-	-	Y14H, N40C, N41D, N68G, K69R, K70R, I89V

[a]Initiated ART five years after study entry (RAL + DRV/r); [b]Initiated ART in the last year of the study (TDF + FTC + RAL); [c]Initiated ART in the second year of the study (AZT + 3TC + SQV/r); changed to ABC + 3TC + RAL two years later and then to AZT + 3TC + RAL

Table S8. Drug resistance mutations of each participant at baseline and year 8

Participant ID	Baseline		Year 8	
	Major mutations	Accessory mutations	Major mutations	Accessory mutations
	(n. of mutated clones/ n. of clones)			
**ART-Experienced**				
**PTHSM1**	I84V (1/6)	None (0/6)	None (0/7)	None (0/7)
**PTHSM2**	None (0/8)	None (0/8)	NA	NA
**PTHSM3**	I84V (10/10), L90M (10/10)	I64V (10/10), V71I (10/10), L99F (10/10)	NA[d]	NA[d]
**PTHSM4**	I54M (2/2), I82F (2/2), L90M (2/2)	V71I (2/2), A73G (2/2)	NA[e]	NA[e]
**PTHSM5**	None (0/9)	None (0/9)	NA	NA
**PTHSM6**	None (0/6)	None (0/6)	None (0/1)	None (0/1)
**PTHSM7**	None (0/3)	None (0/3)	NA	NA
**PTHSM8**	None (0/6)	None (0/6)	NA	NA
**PTHSM9**	None (0/9)	L99F (1/9)	NA	NA
**PTHSM10**	None (0/6)	None (0/6)	NA	NA
**PTHSM11**	I54M (1/2), I82F (1/2)	V10I (2/2)	I54M (2/3), I82F (3/3), L90M (2/3), V47A (1/3)	V10I (3/3), V33I (1/3), V71I (1/3), F85L (1/3)
**PTHSM12**	None (0/9)	None (0/9)	I54M (1/3), I84V (1/3)	V71I (1/3)
**PTHSM13**	None (0/6)	None (0/6)	None (0/9)	None (0/9)
**PTHSM27**	None (0/7)	A73G (1/7)	None (0/3)	None (0/3)
**ART- naïve**				
**PTHSM14[a]**	L90M (4/11)	V10I (1/11), V71I (3/11)	None (0/3)	None (0/3)
**PTHSM15**	None (0/10)	None (0/10)	NA	NA
**PTHSM16**	None (0/1)	None (0/1)	NA	NA
**PTHSM17**	None (0/9)	None (0/9)	NA	NA
**PTHSM18[b]**	None (0/3)	None (0/3)	None (0/1)	V10I (1/1)
**PTHSM19**	None (0/9)	None (0/9)	NA	NA
**PTHSM20**	None (0/9)	None (0/9)	NA	NA
**PTHSM21**	None (0/10)	N88D (1/10)	NA	NA
**PTHSM22**	None (0/9)	V71I (9/9)	NA	NA
**PTHSM23**	I84V (1/10)	None (0/10)	NA	NA
**PTHSM24**	None (0/3)	None (0/3)	NA	NA
**PTHSM25**	L90M (1/8)	None (0/8)	NA	NA
**PTHSM26[c]**	None (0/6)	None (0/6)	None (0/3)	None (0/3)

[a]Initiated ART five years after study entry (RAL + DRV/r); [b]Initiated ART in the last year of the study (TDF + FTC + RAL); [c]Initiated ART in the second year of the study (AZT + 3TC + SQV/r); changed to ABC + 3TC + RAL two years later and then to AZT + 3TC + RAL; [d]died in the beginning of year 3 (January 2009); [e]died in February 2009; NA- not available

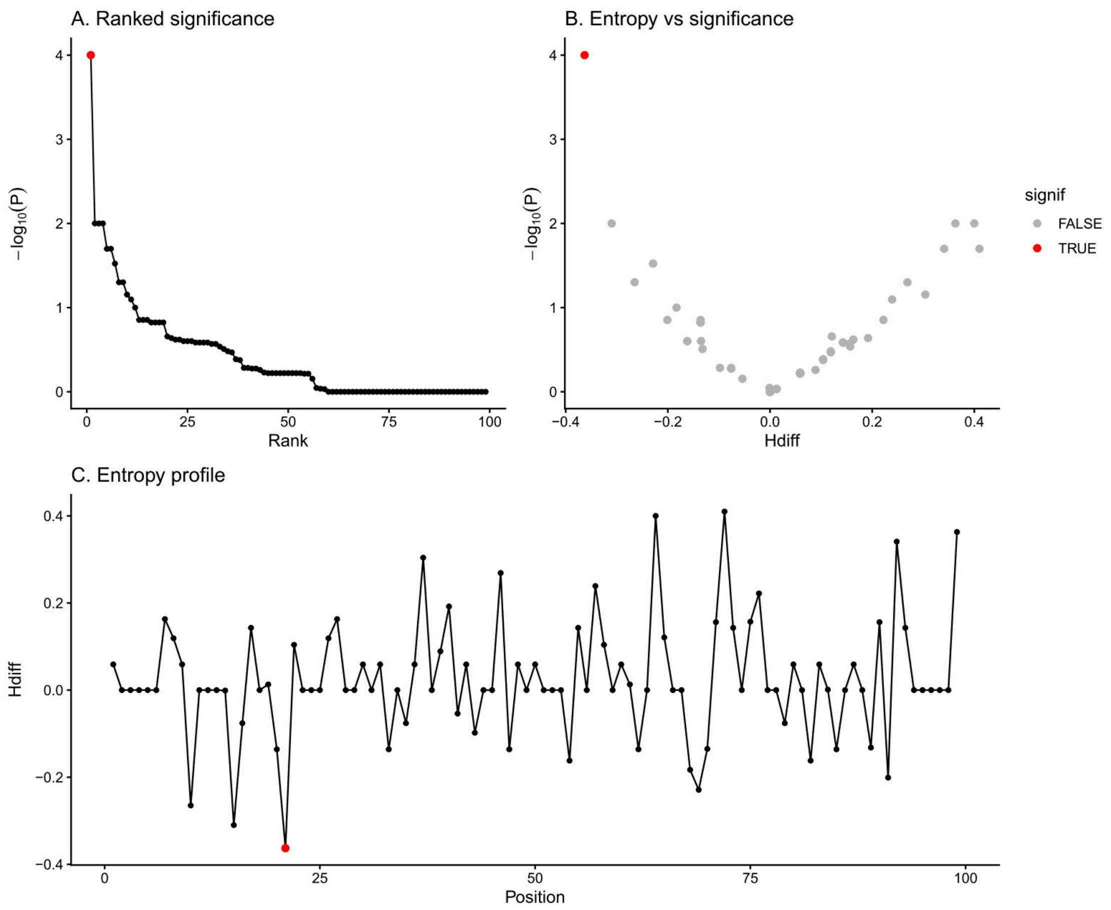

Figure S1. Site-specific entropy differences in HIV-2 protease sequence of ART treated individuals at baseline (background dataset) and study end (query dataset). (A) Ranked distribution of statistical significance across amino acid positions, shown as −log10(empirical P-values) derived from a permutation-based null model (10,000 randomizations per site); (B) Relationship between entropy difference (Hdiff) and statistical significance across all positions; (C) Position-wise entropy profile highlighting variability across the HIV-2 PR sequence.
